# Heterogeneity and New Epitopes of Hepatitis C Virus Genotype 4

**DOI:** 10.5812/hepatmon.10521

**Published:** 2013-08-01

**Authors:** Moataza H. Omran, Wael Nabil, Samar S. Youssef, Mervat El-Sayed, Mostafa K. El Awady

**Affiliations:** 1 Microbial Biotechnology Department, Genetic Engineering Division, National Research Centre, Dokki Cairo, Egypt; 2 Chemistry Department, Faculty of Science, Cairo University, Dokki Cairo, Egypt

**Keywords:** Hepacivirus, Phylogenetic Analysis, Polysaccharides, Epitopes

## Abstract

**Background:**

Hepatitis C virus (HCV) was found to have a major role in human liver disease by its ability to face the host-cell defenses and the immune system. Heterogeneity of HCV was the key for its adaptation to its host and represented a significant hurdle for the development of both effective vaccines as well as for novel therapeutic interventions.

**Objectives:**

Due to the heterogeneity of HCV virus because of both high replication and high mutation rate in vivo, this study was conducted to analyze different isolates of Egyptian patients of genotype 4, of the most mutant regions of the virus (E1 and E2) as they played an important role in viral persistence by escaping from the immune system of the host body.

**Patients and Methods:**

This study was conducted through PCR amplification of E1 and E2 regions, sequencing and phylogenetic analysis, calculating synonyms and non-synonyms substitutions, finding the possible glycosylation sites and different epitope domains.

**Results:**

The present work figured out that the heterogeneity of the quasispecies of our local strains 4a was high showing up 15% diversity. This study also showed four glycosylation sites that play an important role in the entry of the virus and protein folding. Besides, different epitpoes were identified in different regions of the E1 and E2 domains; a finding which would help in determining the neutralizing and non- neutralizing antibodies.

**Conclusions:**

This study would help in understanding the driving forces of genetic diversity and would be fundamental for representing potential candidate targets for antibodies and the development of vaccine trials.

## 1. Background

Hepatitis C virus (HCV) infection is a serious health problem that affects people worldwide. It was figured outthat around 25% of Egyptians are infected by the virus and only 20% of the acutely infected patients might have spontaneous viral clearance. Most of the patients who had chronic hepatitis might then progress to liver cirrhosis and hepatocellular carcinoma. Current therapies of interferon + ribavirin for HCV of genotype 4 were not successful and did not exceed more than 50% and thus finding a cure for this disease is of great importance (1, 2). Hepatitis C virus (HCV) is a positive-stranded RNA molecule of about 9.6 Kb. It consists of large polyprotein of about 3000 amino acids. this protein consists of structural proteins; the core and the envelope, besides non-structural ones such as: NS3, NS4A, NS4B, NS5A and NS5B. These HCV proteins have several cellular functions besides their major function in viral replication (3). Accumulation of mutations during HCV replication leads to its significant genetic heterogeneity. The most heterogeneous parts of the genome are envelope proteins (E1 and E2) while the most conserved regions are parts of the 5'UTR and the terminal 3'UTR , while the core region was semi conserved (4). It had been found that both core and envelope proteins play a major role in various stages of the of the virus' life cycle: including cell entry, uncoating and virion assembly suggested that the core region with envelope proteins have numerous functional activities. These regions were found to play an important role in viral persistence by escaping from the the host's immune system (5). Hepatitis C virus (HCV) E1 and E2 glycoproteins had a high degree of variability which made the virus gained different phenotypic traits; as do alterations in receptor-binding affinity and in immune recognition and escape. It was found that HVR1 had high genetic diversity especially in patients with persistent viremia. Moreover, the rate of non-synonymous substitutions were predominated within the HVR1 region and gradually increased, compared to that in the E1 and E2 regions outside HVR1 (6).

## 2. Objectives

Disease course and antiviral therapy response are affected by a multiplicity of host and viral factors that are under extensive research worldwide. Certain viral genotypes, subtypes and quasispecies are linked to disease progression, however, other studies showed the contrary, thus, due to the heterogeneity of HCV with both high replication and mutation rate in vivo, this study was conducted to analyze different isolates of Egyptian patients in the most mutable (mutation prone) regions of the virus (E1 and E2). These data would be fundamental for the development of different trials for vaccine in Egypt.

## 3. Patients and Methods

### 3.1. Patients

This study was done on Egyptian patients infected with Hepatitis C virus (HCV). This work obeys ethical guidelines and an informed consent was obtained from each patient incorporated in the study. Patients in this study were subjected to the following:

Full history taking, clinical and laboratory examinations where the mean ± SD of age was (47.5 ± 5.6) years old. Males percentage in this study was (80%) while females were (20%). An elevation in liver enzymes (ALT and AST) was observed in 75% of the cases studied.

### 3.2. HCV RNA Tests

These include qualitative HCV nested RT-PCR, genotyping of HCV RNA genome. Methods used for these assays were previously described as follows; nested RT-PCR (7) and HCV genotyping (8).

### 3.3. Nested PCR Amplification of HCV E1-E2 Regions Using Specific Primers

Primers' sequences were as follows:

F1: 5' CACTGGACYACBCARGA NTGYAA 3'

(Where Y is C or T; B is G, T, or C; R is G or A; and N is A, C, G, or T)

F2: 5' ATGGCNTGGGAYATGATGATGAA 3'

(Where N is A, C, G, or T; and Y is C or T)

R1: 5' TTGGTGAACCCDGTRCYRTTCA 3'

(Where D is G, A, or T; R is G or A; and Y is C or T)

R2: 5' TGAACCCDGTRCYRTTCATTCA 3'

(Where D is G, A, or T; R is G or A; and Y is C or T).

R3: 5' AGGAAGACATCNGTNTCRTTCTC 3'

(Where N is A, C, G, or T and R is G or A)

cDNA synthesis and first round PCR were performed simultaneously using RT-PCR beads (Amersham Biosciences, USA), for each sample 6µl of RNA were added with 5µl of each of the following specific primers (R1, R2 and F1). The reverse transcription Thermal cycling was 30 minutes at 42°C followed by 5 minutes at 94°C. First PCR round was: 40 cycles for 40 seconds at 94°C, 20seconds at 53°C and 1 minute at 72°C, then final extension for 7 minutes at 72°C. For the second amplification specific primers (F2 and R3) were used. The Thermal cycling protocol was 9 minutes at 95° C then 35 cycles for 40 seconds at 94° C, 10seconds at 53.5° C and 1 minute at 72° C, then final extension for 7 minute at 72° C. 10μl of the each amplicon were electrophoresed on a 2% agarose gel.

### 3.4. Cloning of DNA Fragments

The pGEM-T Vector system (Promega, USA) was used to clone purified PCR products. The ligated plasmids were transformed into highly efficient competent cells namely JM 109. Plasmid DNA was isolated using plasmid miniprep method using (Wizard plus Minipreps DNA Purification System Kit, Promega-USA).

### 3.5. Sequencing and Phylogenetic Analysis

Samples further underwent sequence analysis in the Automated Sequencer “ABI Prism 310 Genetic Analyzer”. The sequences were aligned with the consensus sequences of genotype 4 that were retrieved from Gene Bank using the program ClustalX implemented in the Bioedit package. The PHYLIP suit of programs, version 3.572 (9) was used to generate the Phylogenetic tree.

The DNADIST program was used to calculate the Genetic distance matrices which were then used to generate a tree using NEIGHBOR and the SEQBOOT program. Reference sequences of different HCV genotypes were retrieved from Gene Bank.

### 3.6. Computer-Assisted Analysis of N Glycosylation Sites and Epitope Prediction

N glycosylation sites were predicted using the on-line prediction server NetNGlyc version 1.0.Epitopes. Prediction of T-cell was performed using the on-line software SYFPEITHI (version 1) (10).

### 3.7. Secondary Structural Prediction

All the E1/E2 sequence of 28 Egyptian clones was uploaded to the internet server, and the secondary structures waere predicted automatically using SOPMA library.

## 4. Results

### 4.1. Clinical Data

All the Egyptian patients infected with Hepatitis C Virus (HCV) fulfilled the criteria for being covered by the national health program for treating viral hepatitis and they were free from co-infection with HBV, HIV or Schistosomiases, no thyroid dysfunction, no uncontrolled diabetes mellitus, no obesity, no history of long term drug or alcohol intake.

### 4.2. Sequencing and Phylogenetic Analysis

Nucleotide and amino acid (a.a) sequences of the amplified HCV E1/E2 28 Egyptians clones were determined and aligned with sequences of HCV prototypes and other genotypes taken from Gene Bank.

**Figure 1.**
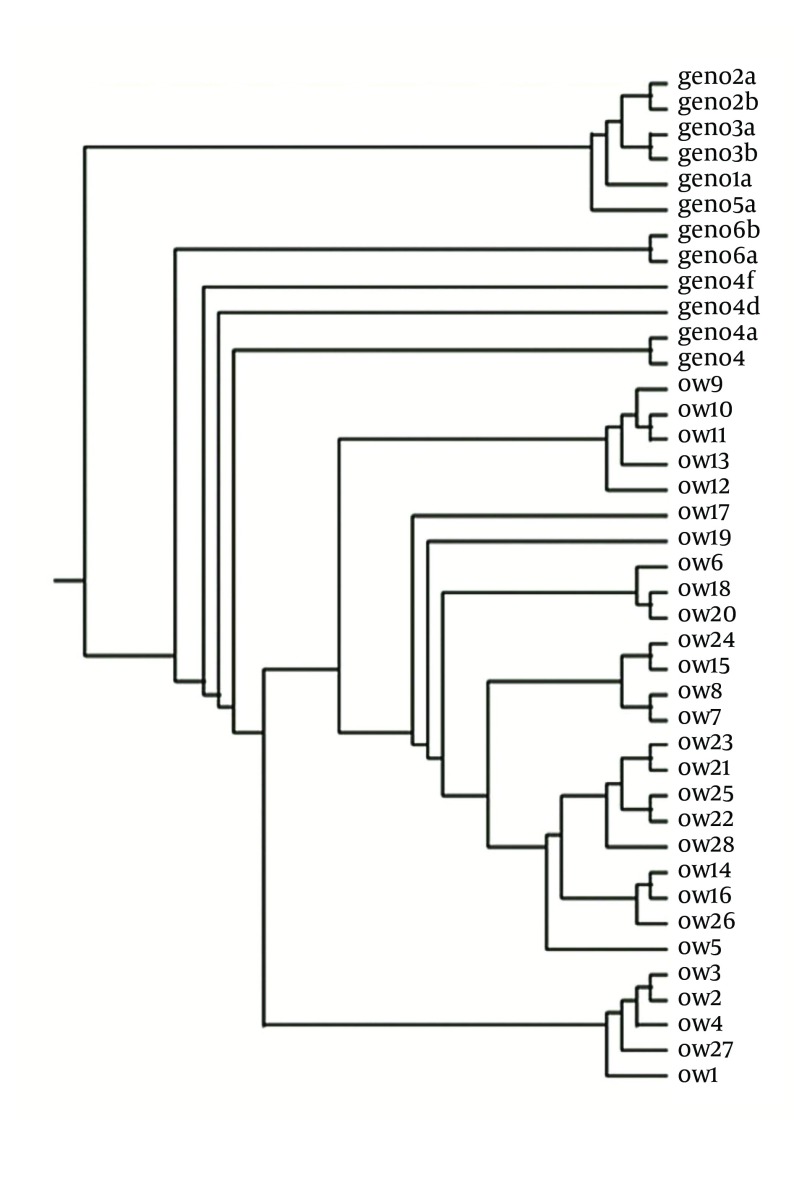
Phylogenetic Tree of HCV E1-E2 Regions of 28 Egyptian Strains The sequence of HCV E1/E2 Egyptian clones unraveled in this study had been submitted to Gene Bank under the following accession numbers: OW1(HM596343), OW2(HM596344), OW3(HM596345), OW4(HM596346), OW5(HM596347), OW6(HM749053), OW7(HM596348), OW8(HM596349), OW9(HM596350), OW10(HM596351),OW11(HM596352), OW12 (HM623431), OW13 (HM623432), OW14 (HM623433), OW15 (HM623434),OW16 (HM623435), OW17 (HM623436), OW18 (HM623437), OW19 (HM623438), OW20 (HM623439),OW21 (HM623440), OW22 (HM623441), OW23 (HM749054), OW24 (HM749055), OW25 (HM749056),OW26 (HM749057), OW27 (HM749058), OW28 (HM749059).

The phylogenetic tree was generated using the reference sequences of HCV prototype (HCp) and genotypes of HCV (1a, 1b, 2a, 2b, 3a, 3b, 4a, 4c, 4f, 5a and 6a) retrieved from the Gene bank and the sequence data of the 28 Egyptian clones whose accession number in the Gene Bank is shown in Figure 1.

### 4.3. Multiple Alignment

**Figure 2.**
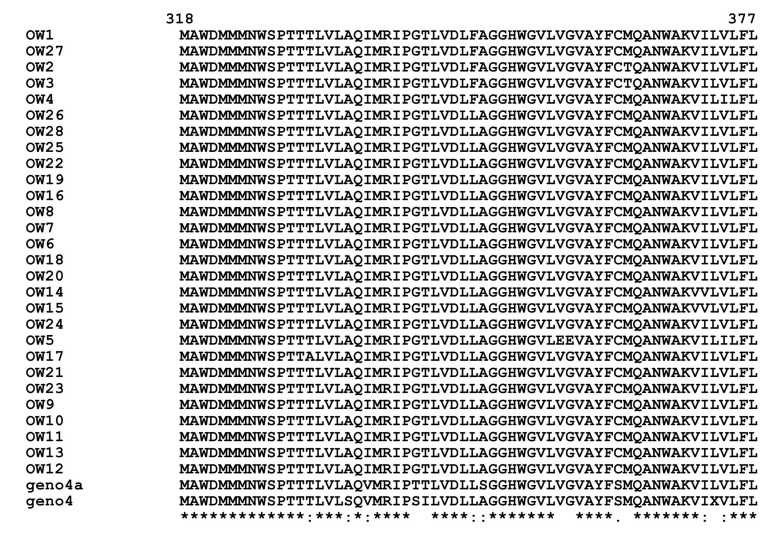
Multiple Alignment for E1/E2 Amino Acid Sequences Means that all the sequences are identical in in the alignment. showed the conserved substitutions. “showed the semi-conserved substitutions. The space means that substitutions are observed.

**Figure 3.**
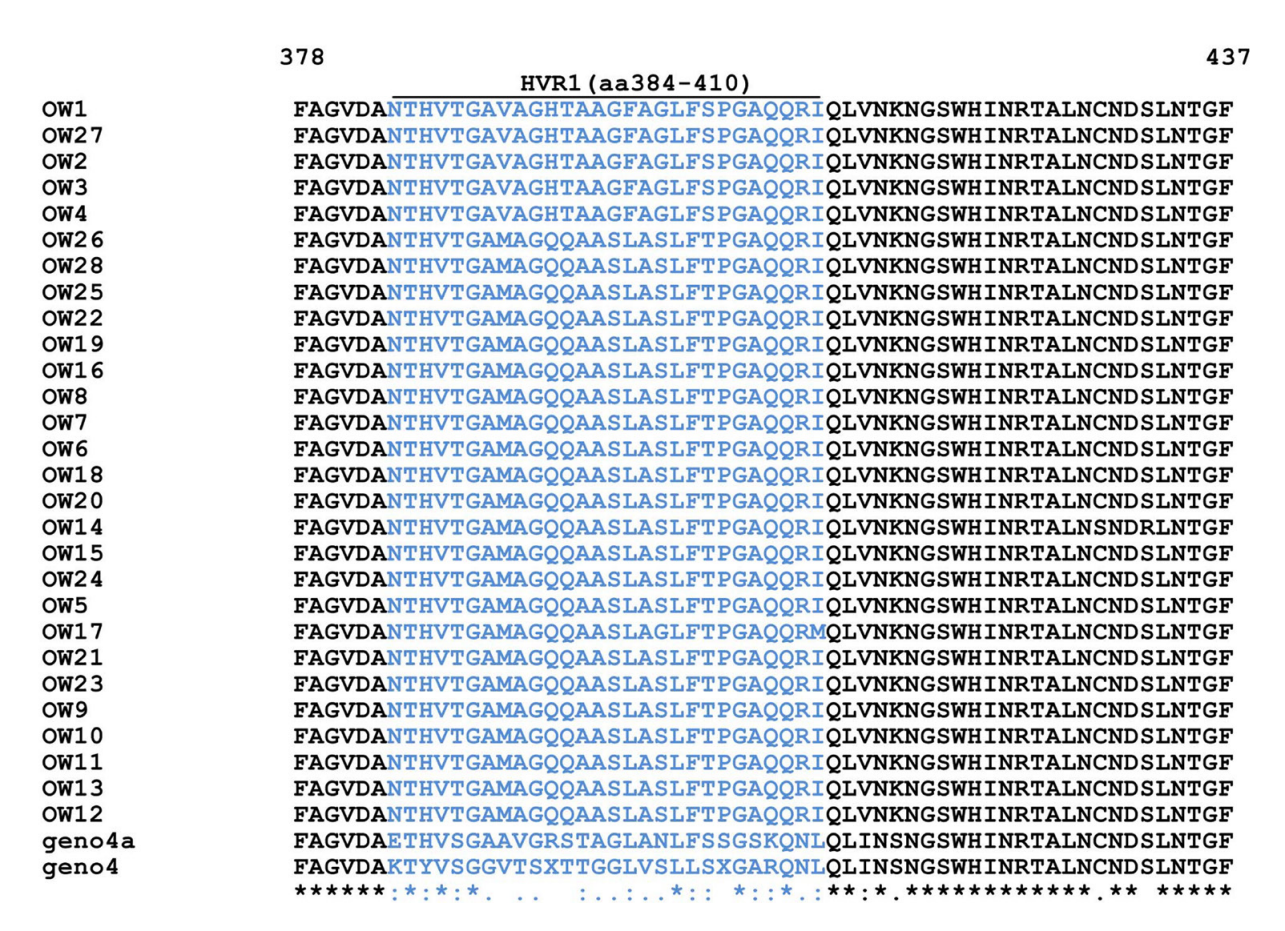
Multiple Alignment for E1-E2 (HVR1) Amino Acids of 28 Egyptian Local Strains with Prototype 4a Means that all the sequences are identical in in the alignment. showed the conserved substitutions. “showed the semi-conserved substitutions. The space means that substitutions are observed.

**Figure 4.**
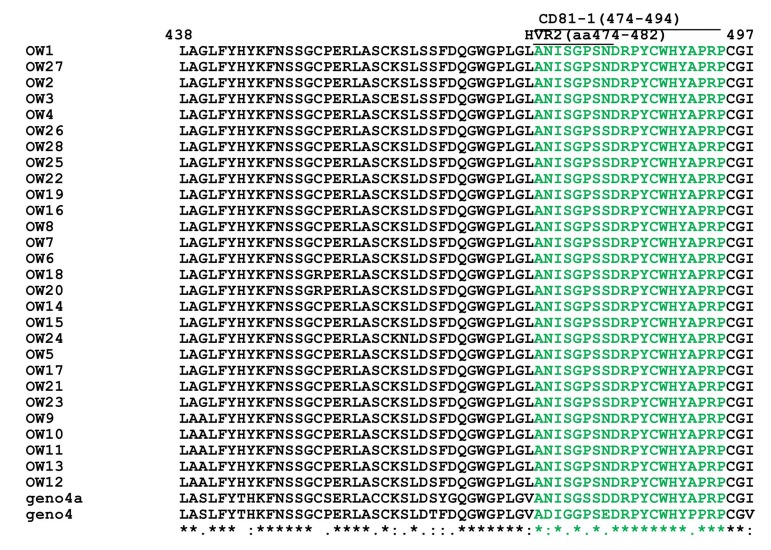
Multiple Alignment for E1-E2 (HVR2 and CD81-1) Amino Acids of 28 Egyptian local strains with prototype 4a "Means that all the sequences are identical in in the alignment. "showed the conserved substitutions. “showed the semi-conserved substitutions. The space means that substitutions are observed.

**Figure 5.**
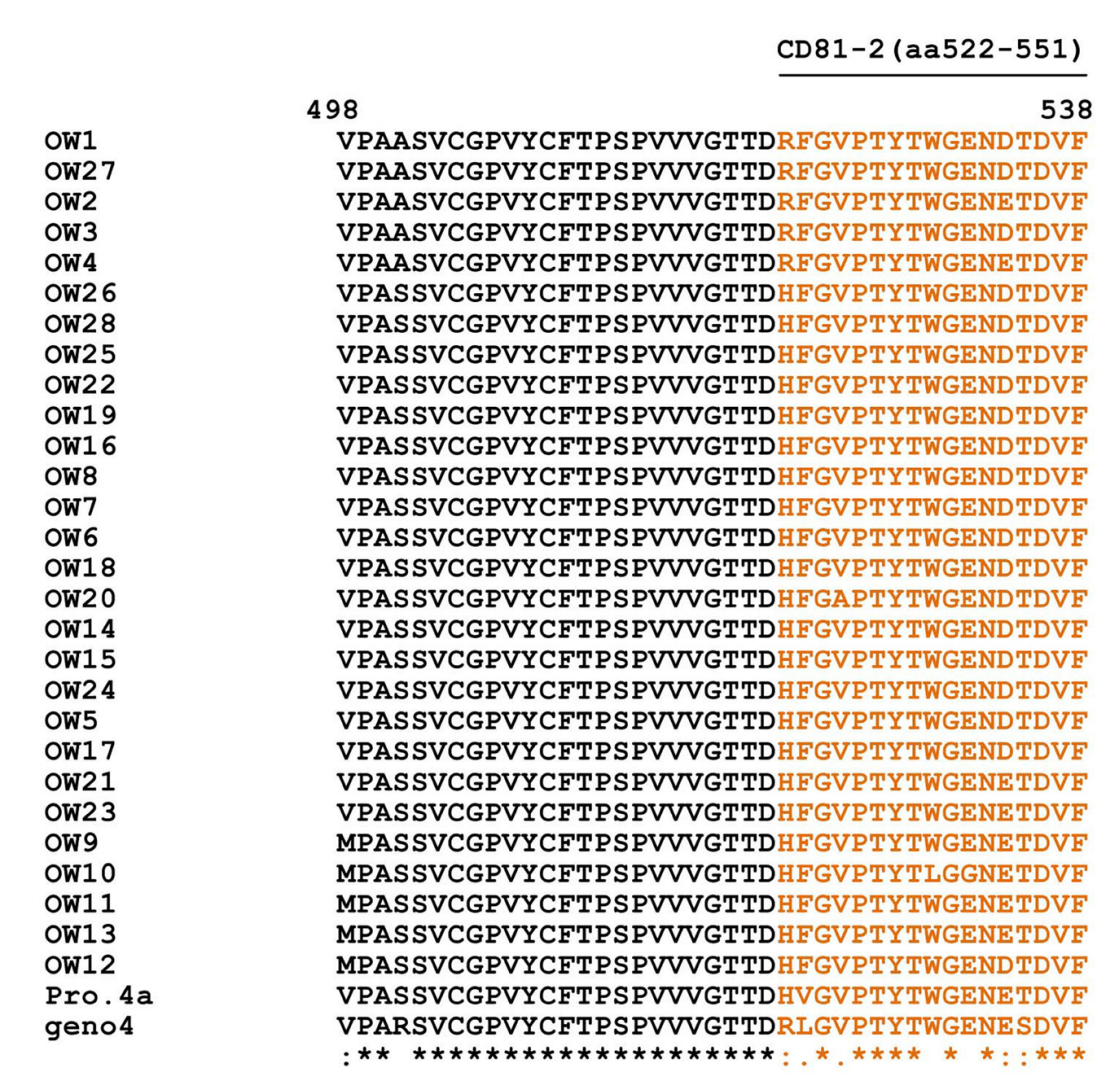
Multiple Alignment for E1-E2 (CD81-2) Amino Acids of 28 Egyptian Local Strains With Prototype 4a "Means that all the sequences are identical in in the alignment. ”showed the conserved substitutions. “showed the semi-conserved substitutions. The space means that substitutions are observed.

Multiple alignment of E1/E2 amino acid sequences of 28 Egyptian local strains with prototype 4a and genotype 4 is shown in Figure 2 and in HCV E1/ E2 Domains for HVR1 (aa384-410) (Figure 3), HVR2 (aa474-482) and CD81-1(474-494) (Figure 4) and CD81-2(aa522-551) (Figure 5).

### 4.4. Probability of Glycosylation Sites

In the present study, all of the isolates showed four different N-glycosylation sites, except for OW14 (HM623433) which showed only three sites. Potential glycosylation sites verge at positions 100, 113, 159, and 216. The probability of the potential glycosylation sites in the 28 Egyptian strains are shown in (Table 1), glycosylation sites at positions 100, 113, 159, and 216 correspond to amino acids 418, 431, 477 and 534 in HCV 4a.

**Table 1.** Probability of the Potential Glycosylation Sites In The 28 Egyptian Strains

Isolate	Probability of glycosylation position							No. of sites
	8	100	106	113	131	159	216	
**OW1**	---	+^a^	-a	+	-	+	+	4
**OW2**	---	+	-	+	-	+	+	4
**OW3**	---	+	-	+	-	+	+	4
**OW4**	---	+	-	+	-	+	+	4
**OW5**	---	+	-	+	-	+	+	4
**OW6**	---	+	-	+	-	+	+	4
**OW7**	---	+	-	+	-	+	+	4
**OW8**	---	+	-	+	-	+	+	4
**OW9**	---	+	-	+	-	+	+	4
**OW10**	---	+	-	+	-	+	+	4
**OW11**	---	+	-	+	-	+	+	4
**OW12**	---	+	-	+	-	+	+	4
**OW13**	---	+	-	+	-	+	+	4
**OW14**	---	+	-	Not predicted	-	+	+	3
**OW15**	---	+	-	+	-	+	+	4
**OW16**	---	+	-	+	-	+	+	4
**OW17**	---	+	-	+	-	+	+	4
**OW18**	---	+	-	+	-	+	+	4
**OW19**	---	+	-	+	-	+	+	4
**OW20**	---	+	-	+	-	+	+	4
**OW21**	---	+	-	+	-	+	+	4
**OW22**	---	+	-	+	-	+	+	4
**OW23**	---	+	-	+	-	+	+	4
**OW24**	---	+	-	+	-	+	+	4
**OW25**	---	+	-	+	-	+	+	4
**OW26**	---	+	-	+	-	+	+	4
**OW27**	---	+	-	+	-	+	+	4
**OW28**	---	+	-	+	-	+	+	4
**Geno 4a**	---	++	-	+	-	+	+	4

^a^ +, Probability of glycosylation sites; -, Glycosylation sites not present

### 4.5. Synonymous and Non-Synonymous Substitution Rates

Calculation of synonymous and non-synonymous substitution rates in 28 Egyptians clones compared to prototype 4a (Y11604) are shown in (Table 2). 

**Table 2.** Synonymous and Non-Synonyms Rates

Compare		Sequences	names	Sd^a^	Sn^a^	S^a^	No.^a^	Ps^a^	Pn^a^	Ds^a^	Dn^a^	ds/dn^a^	ps/pn
**0**	1	Y11604 (4a)	OW1	58.0000	44.0000	152.1667	513.8333	0.3812	0.0856	0.5323	0.0909	5.8540	4.4512
**0**	2	Y11604 (4a)	OW2	56.0000	44.0000	152.1667	513.3333	0.3668	0.0857	0.5037	0.0910	5.5335	4.2935
**0**	3	Y11604 (4a)	OW3	56.0000	46.0000	152.1667	513.3333	0.3668	0.0896	0.5037	0.0954	5.2777	4.0934
**0**	4	Y11604 (4a)	OW4	57.0000	44.0000	152.0000	514.0000	0.3750	0.0856	0.5199	0.0909	5.7193	4.3807
**0**	5	Y11604 (4a)	OW5	49.0000	46.0000	149.6667	516.3333	0.3274	0.0891	0.4302	0.0948	4.5363	3.6749
**0**	6	Y11604 (4a)	OW6	48.0000	43.0000	150.5000	515.5000	0.3189	0.0834	0.4154	0.0884	4.6972	3.8235
**0**	7	Y11604 (4a)	OW7	48.0000	43.0000	150.5000	515.5000	0.3189	0.0834	0.4154	0.0884	4.6972	3.8235
**0**	8	Y11604 (4a)	OW8	48.0000	43.0000	150.5000	515.5000	0.3189	0.0834	0.4154	0.0884	4.6972	3.8235
**0**	9	Y11604 (4a)	OW9	53.0000	43.0000	150.5000	516.0000	0.3533	0.0833	0.4777	0.0883	5.4081	4.2259
**0**	10	Y11604 (4a)	OW10	56.0000	45.0000	150.6667	515.3333	0.3717	0.0873	0.5133	0.0928	5.5285	4.2564
**0**	11	Y11604 (4a)	OW11	54.0000	43.0000	150.0000	516.0000	0.3600	0.0833	0.4904	0.0883	5.5395	4.3200
**0**	12	Y11604 (4a)	OW12	55.0000	44.0000	150.3333	515.6667	0.3659	0.0853	0.5018	0.0906	5.5395	4.2877
**0**	13	Y11604 (4a)	OW13	51.0000	43.0000	150.0000	516.0000	0.3400	0.0833	0.4529	0.0883	5.1274	4.0800
**0**	14	Y11604 (4a)	OW14	48.0000	46.0000	150.8333	515.1667	0.3182	0.0893	0.4141	0.0951	4.3561	3.5640
**0**	15	Y11604 (4a)	OW15	48.0000	44.0000	150.6667	515.3333	0.3186	0.0854	0.4148	0.0906	4.5755	3.7313
**0**	16	Y11604 (4a)	OW16	51.0000	43.0000	150.5000	515.5000	0.3389	0.0834	0.4509	0.0884	5.0988	4.0625
**0**	17	Y11604 (4a)	OW17	49.5000	45.5000	150.5000	515.5000	0.3289	0.0883	0.4329	0.0939	4.6101	3.7264
**0**	18	Y11604 (4a)	OW18	51.0000	44.0000	150.8333	515.1667	0.3381	0.0854	0.4495	0.0907	4.9573	3.9588
**0**	19	Y11604 (4a)	OW19	51.0000	43.0000	150.5000	515.5000	0.3389	0.0834	0.4509	0.0884	5.0988	4.0625
**0**	20	Y11604 (4a)	OW20	53.0000	45.0000	150.8333	515.1667	0.3514	0.0874	0.4741	0.0929	5.1044	4.0227
**0**	21	Y11604 (4a)	OW21	49.0000	42.0000	150.5000	515.5000	0.3256	0.0815	0.4270	0.0862	4.9510	3.9961
**0**	22	Y11604 (4a)	OW22	48.0000	43.0000	150.5000	515.5000	0.3189	0.0834	0.4154	0.0884	4.6972	3.8235
**0**	23	Y11604 (4a)	OW23	49.0000	42.0000	150.5000	515.5000	0.3256	0.0815	0.4270	0.0862	4.9510	3.9961
**0**	24	Y11604 (4a)	OW24	50.0000	44.0000	150.8333	515.1667	0.3315	0.0854	0.4375	0.0907	4.8252	3.8812
**0**	25	Y11604 (4a)	OW25	48.0000	43.0000	150.5000	515.5000	0.3189	0.0834	0.4154	0.0884	4.6972	3.8235
**0**	26	Y11604 (4a)	OW26	50.0000	43.0000	150.5000	515.5000	0.3322	0.0834	0.4388	0.0884	4.9628	3.9828
**0**	27	Y11604 (4a)	OW27	53.0000	44.0000	152.1667	513.8333	0.3483	0.0856	0.4683	0.0909	5.1501	4.0675
**0**	28	Y11604 (4a)	OW28	50.0000	43.0000	150.5000	515.5000	0.3322	0.0834	0.4388	0.0884	4.9628	3.9828

^a^ Abbreviations: Sd, the number of observed synonymous substitutions; Sn, the number of observed non-synonymous substitutions; S, the number of potential synonymous substitutions (the average for the two compared sequences); No., the number of potential non-synonymous substitutions (the average for the two compared sequences); ps, the proportion of observed synonymous substitutions =Sd/S; pn, the proportion of observed non-synonymous substitutions = Sn/N; ds/dn, the ratio of synonymous to non-synonymous substitutions

### 4.6. Prediction of the Epitope Domain

Six classes of epitopes were observed in the 28 Egyptian strains, first class was (H2-Db nonamers), second class (H2-Kd nonamer), third class (HLA-A*26), fourth class (HLA-B*1402), fifth class (HLA-B*2705) and sixth class (HLA-A*0201). The prediction of the epitope domains in E1-E2 regions of 28 Egyptian strains was shown in Table 3. 

**Table 3.** Prediction of the Epitope Domains in the E1-E2 Regions of the 28 Egyptian Strains

Score	Epitope Domains of Egpytian stains	Position^a^	Epitope Domains classes
	OW1…..OW28		H2-Db
	4a		Nonamer
**24**	S W H I N R T A L	420	
	OW1…..OW28		H2-kD
	4a		Nonamer
**24**	H Y A P R P C G I	490	
	OW1…..OW3		HLA-A*26
	OW6…..OW28		Nonamer
	4a		
**26**	K V I L V L F L F	371	
	OW4,OW5		
**28**	K V I L I L F L F	371	
	OW1…..OW28		HLA-B*1402
	4a		Nonamer
**25**	E R L A S C K S L	455	
	OW1…..OW23		HLA-B*2705
	OW25…..OW28		Nonamer
**24**	E R L A S C K S L	455	
	OW24		
**25**	E R L A S C K N L	455	
	4a		
**23**	E R L A C C K S L	455	
	OW1…..OW28		HLA-A*0201
**26**	R I P G T L V D L	340	Nonamer
	4a		
**24**	R I P T T L V D L	340	
	OW1…..OW4		
	OW27		
**19**	L F A G G H W G V	348	
	OW5…..OW26		
	OW28		
**29**	L L A G G H W G V	348	
	4a		
**27**	L L S G G H W G V	348	
	OW1…..OW3		
	OW6…..OW9		
	OW12…..OW28		
	4a		
**21**	L V L F L F A G V	374	
	OW4,OW5		
	OW10,.OW11		
**26**	L I L F L F A G V	374	

^a^ Only scores > 23 are considered

### 4.7. Secondary structural Results

The data have showen differences between different HCV Egyptian isolates and the prototype 4a in the protein secondary structure.. In our isolates, 51 aa. of the 221 aa. scored an alpha helix secondary structures, whereas in the prototype 4a this score was observed for 47aa of the 221 aas. Extended strand (Ee) in our Egyptian isolates recorded (60aa. up to 64aa.) while the prototype 4a recorded (56aa). Beta turn (Tt) in our Egyptian isolates recorded up to (17aa.) and in prototype 4a recorded (13aa.) and random coil (Cc) in our Egyptian isolates recorded (91aa. up to 102aa.) and in prototype 4a recorded 105aa.

## 5. Discussions

Egypt is considered the best model site for vaccine trials because the prevalence of HCV infections is high and extreme heterogeneity of HCV found at all levels. The development of an effective vaccine is influenced by many factors like proper folding of structural proteins, the eliciting of a neutralizing immune response and more importantly the viral heterogeneity due to its great role in disease progression therapy. This leads us to study in this research the most mutation prone regions of the virus (E1 and E2) in different isolates of Egyptian patients. Within the infected host a single isolate comprises many millions of both different and closely related sequences called quasispecies that provided a large store of different viral variants that had significant clinical effects helping the virus to escape the immune mechanism (11). The present work established that the heterogeneity of the quasispecies of our local strains of E1-E2 was high showing up to 15% diversity that might have important consequences during a transmission route depending on the number of transmitted viral RNA copies that might be limited and not representing the true complexity of the sequence diversity of the donor (12). However, this bottleneck could also be interpreted as selection of the optimal strain in the new host during the earliest infection events (13).

Accumulation of mutations during viral replication was either silent synonymous that had no effect on the sequence of the viral protein, but sometimes could affect the secondary structure of RNA. The other one was called non-synonymous mutations that caused changes in protein sequence that led to the production of different newly defective viral variants that could be lethal and aggressive (14, 15). The presented data in this research were interesting in light of analyzing the substitution rates of synonymous and non-synonymous mutations of the HCV E1-E2 local strains as the average number of potential synonymous substitutions in the sequences of the local isolates were 150.7 while those of the potential non-synonymous substitutions were 515 (Table 2).Thus, the number of non-synonymous mutations exceeded the number of synonymous mutations three times that warns us to the emergence of aggressive variants as these mutations led to the production of defective viral particles in the Egyptian population and could be lethal. 

By having the sequencing data from the core gene of HCV genotype 4 of the Egyptian strains that had showed genetic relevance to HCV types 1 (16), in addition to the high number of the non-synonymous mutations of E1-E2 found in this study, suggests that the evaluation of the genotype 4 could happen in the same patient through multiple mutational ways of type 4 into genotype 1 which is closely related to it. Moreover, this might explain why the majority of the Egyptians, about 70%, did not respond to interferon therapy. In accordance to (17) who reported that genetic variations of NS5 and E2 proteins of HCV genotype 1 would inhibit the double stranded (ds) RNA–dependent protein kinase that is involved in the cellular antiviral response induced by interferon.

It was reported that N-linked glycans in viral envelope proteins could play a major role in the viral entry, or in modulating the response to treatment (18, 19). In the present study, all of the 28 Egyptian isolates showed four different glycosylation sites at positions 418, 431, 477 and 534 with the exception of clone OW14 (HM623433) which lacked the glycosylation site at position 431 (Table 1). Those glycans were found to play an important role in HCV entry and/or protein folding (20). In this study, two of the glycans (477, 534) were in the HVR2 and in the binding site of CD81 indicating that these regions were major targets for neutralizing antibodies. Furthermore, those glycans on E2 that attached to the binding site of CD81 might reduce the sense of HCV pp (HCV pseudo-particles) to antibody neutralization and consequently reduce the access of CD81 to its E2 binding site (21). On the other hand, (22) found that the absence of glycans at positions: 196, 305, 556 or 623 strongly influences the incorporation of HCV glycoproteins into HCVpp (HCV pseudo-particles), suggesting that these glycans are essential for protein folding. This was long-established by our data, as it lacked these glycans, suggesting the effect of their absence in viral entry. 

It was found that most of the induced antibodies had no antiviral activity. This was because they either targeted against useless epitopes that had no role in virus entry or they were produced by degraded intracellular proteins released from dying cells (23). Only “neutralizing antibodies” succeeded in both targeting the exposed epitopes and neutralizing the viral infection by controlling or preventing it (24). Also, it was known that identification of many CTL epitopes is required especially for the development of a successful immunogenic vaccine.

This work observed six classes of epitopes shown in the results section (Table 3). The present data showed that the class (HLA-A*0201) exhibits three epitopes (Table 3), two of them could be utilized as having score more than the prototype 4a. The first one at (aa 340-348) and the second one at (aa 348-356) where 23 clones had mutations of score 29 more than that prototype 4a which is of score 27 indicating decreasing affinity of binding. However, (25) represented (HLA-A0201) an epitope that was not utilized because it did not have a proteasome cleavage site and had mutations that caused a decrease in the score. Also, two clones of the second class of epitope (HLA-A*26) (aa 371-379) had mutations and a score of 28 more than the prototype 4a and these could be used for synthetic HCV peptides. The fifth class (HLA-B*2705) (aa 455-463) has all the 28 Egyptian isolates had score of 24 higher than the score of prototype 4a (23) with the exception of clone (OW24 of score 25) (Table 3). These epitopes could be targeted by neutralizing antibodies and could exhibit broad cross-neutralizing activity among all major genotypes of HCV pp (HCV pseudo-particles) entry (26). Also, these epitopes could have a role in membrane fusion processes and could represent a target for monoclonal antibodies (27, 28). The present work also showed the sixth class (H2-Kd nonamer) (aa 490-498) where all the 28 Egyptian isolates had score of 24 the same as the score of prototype 4a (Table 3) and those epitopes are different from that shown before in former report (25). Those epitopes could be essential for immunoreactivity as the binding site of E2 of CD81 located at aa480–493 and aa544–551 of E2 (29).

Furthermore, our data showed that the secondary structures of the E1-E2 region of our Egyptian HCV strains are different from former report (25). They have conformational and folding structure stronger than that of the prototype 4a. These secondary structures in this study might help the HCV polyprotein to undergo a series of cleavages to form functional viral proteins and guide HCV to hide their epitopes from the immune system and this coincides with former studies (21, 30).

The results of this study demonstrated different spectra of viral strains of HCV E1-E2 isolates showing synonyms and non-synonyms substitutions, finding glycosylation sites and different epitope domains that could be used to produce monoclonal or polyclonal antibodies targeting both linear and conformational epitopes of envelope glycoprotein E2 that have been shown to inhibit cellular binding of HCV-LP (HCV- Leucoplasts), entry of HCVpp (HCV pseudo-particles) and infection of HCVcc (HCV cell culture).

In conclusion, this study is considered as an important research tool for the development of vaccine trials in Egypt and would help in explaining the existing intra-individual variations of patients in response to the existing drugs in the local market.
